# PSO-Based Support Vector Machine with Cuckoo Search Technique for Clinical Disease Diagnoses

**DOI:** 10.1155/2014/548483

**Published:** 2014-05-25

**Authors:** Xiaoyong Liu, Hui Fu

**Affiliations:** ^1^Department of Computer Science, Guangdong Polytechnic Normal University, Guangzhou, Guangdong 510665, China; ^2^School of Business Administration, South China University of Technology, Guangzhou, Guangdong 510640, China

## Abstract

Disease diagnosis is conducted with a machine learning method. We have proposed a novel machine learning method that hybridizes support vector machine (SVM), particle swarm optimization (PSO), and cuckoo search (CS). The new method consists of two stages: firstly, a CS based approach for parameter optimization of SVM is developed to find the better initial parameters of kernel function, and then PSO is applied to continue SVM training and find the best parameters of SVM. Experimental results indicate that the proposed CS-PSO-SVM model achieves better classification accuracy and F-measure than PSO-SVM and GA-SVM. Therefore, we can conclude that our proposed method is very efficient compared to the previously reported algorithms.

## 1. Introduction

Accurate diagnosis and effective treatment of disease are important issues in life science research and have a positive meaning for human health. Recently, medical experts pay more attention to early diagnosis of disease and propose many new methods to deal with disease diagnosis problem. Using machine learning methods to diagnose disease is rapid development of a novel research branch of machine learning. Researchers have applied artificial intelligence and computer technology to develop some medical diagnostic systems, which improve the efficiency of diagnosis and become practical tools.

It is shown that support vector machine has good generalization ability and has been widely used in many research areas, such as signal classification [1], image processing [2], and disease diagnosis [3–6]. Illán et al. [3] showed a fully automatic computer-aided diagnosis (CAD) system for improving the accuracy in the early diagnosis of the AD. The proposed approach is based firstly on an automatic feature selection and secondly on a combination of component-based support vector machine (SVM) classification and a pasting votes technique of assembling SVM classifiers. Sartakhti et al. [4] proposed a novel machine learning method that hybridized support vector machine (SVM) and simulated annealing (SA) for hepatitis disease diagnosis. The obtained classification accuracy of SVM-SA method was 96.25% and it was very promising with regard to the other classification methods in the literature for this problem. The approach proposed by Ramírez et al. [5] was based on image parameter selection and support vector machine (SVM) classification. The proposed system yielded a 90.38% accuracy in the early diagnosis of Alzheimer's disease and outperformed existing techniques including the voxel-as-features (VAF) approach. Abdi and Giveki [6] developed a diagnosis model based on particle swarm optimization (PSO), support vector machines (SVMs), and association rules (ARs) to diagnose erythematosquamous diseases. The proposed model consists of two stages: first, AR is used to select the optimal feature subset from the original feature set. Then, a PSO-based approach for parameter determination of SVM is developed to find the best parameters of kernel function (based on the fact that kernel parameter setting in the SVM training procedure significantly influences the classification accuracy and PSO is a promising tool for global searching).

Support vector machine is a machine learning algorithm based on statistical learning theory and has the strong predictive ability for nonlinear problems. However, SVM prediction performance is closely related to the quality of the selected parameters. Parameter optimization algorithms currently used are particle swarm optimization and genetic algorithms, but these algorithms have their shortcomings and affect the accuracy of disease prediction.

Cuckoo search (CS) is a new swarm intelligent optimization algorithm. Preliminary studies show that cuckoo search algorithm is simple and efficient, easy to implement and has less parameters [7]. Cuckoo search algorithm is able to provide a new method for the SVM parameter optimization. This paper proposes a disease diagnosis model based on cuckoo search, particle swarm optimization (PSO), and support vector machine.

The structure of this paper is as the following.  Section 2 firstly introduces related algorithms, such as support vector machine and cuckoo search, and then presents the novel models, CS-PSO-SVM.  Section 3 gives results of different models in two real disease diagnoses datasets from University of California Irvine Machine Learning Repository. Finally, conclusions are presented in Section 4.

## 2. Methods and Materials

### 2.1. Methods

#### 2.1.1. SVM

Support vector machines [8–10] (SVMs) are a set of related supervised learning methods used for classification and regression. A support vector machine constructs a hyperplane or set of hyperplanes in a high-dimensional space, which can be used for classification, regression, or other tasks. Intuitively, a good separation is achieved by the hyperplane that has the largest distance to the nearest training data points of any class (so-called functional margin), since, in general, the larger the margin, the lower the generalization error of the classifier.

In order to extend the SVM methodology to handle data that is not fully linearly separable, we relax the constraints slightly to allow for misclassified points; the formulation is following (1). This is done by introducing a positive slack variable *ξ*_*i*_, *i* = 1,2,…*L*:

(1) $$\begin{array}{c} x_{i}\cdot w+b\geq +1-\xi_{i}\;\left(y_{i}=+1\right),\\ x_{i}\cdot w+b\leq -1+\xi_{i}\;\left(y_{i}=-1\right) \end{array}$$

which can be combined into

(2) $$\begin{array}{c} y_{i}\left(x_{i}\cdot w+b\right)-1+\xi_{i}\geq 0, \end{array}$$

where *ξ*_*i*_ ≥ 0.

In this soft margin SVM, data points on the incorrect side of the margin boundary have a penalty that increases with the distance from it. As we are trying to reduce the number of misclassifications, a sensible way to adapt our objective function from the previously mentioned is to find

(3)  min⁡ 12||w2||+C∑i=1Lξi s.t.   yi(xi·w+b)−ε+ξi≥0,

where the parameter *C* controls the trade-off between the slack variable penalty and the size of the margin. Reformulating as a Lagrange, which as before we need to minimize with respect to *w*, *b*, and *ξ*_*i*_ and maximize with respect to *α* (where *α*_*i*_ ≥ 0, *u*_*i*_ ≥ 0),

(4) LP≡12||w||2+C∑i=1Lξi −∑i=1Lαi[yi(xi·w+b)−1+ξi]−∑i=1Lμiξi.

Differentiating with respect to *w*, *b*, and *ξ*_*i*_ and setting the derivatives to zero,

(5) ∂LP∂w=0⟹w=∑i=1Lαiyixi,∂LP∂b=0⟹∑i=1Lαiyi=0,∂LP∂ξi=0⟹C=αi+μi.

So, we need to find

(6)  max⁡α [∑i=1Lαi−12αTHα] s.t. 0≤αi≤C, ∑i=1Lαiyi=0.

**Figure 1:**
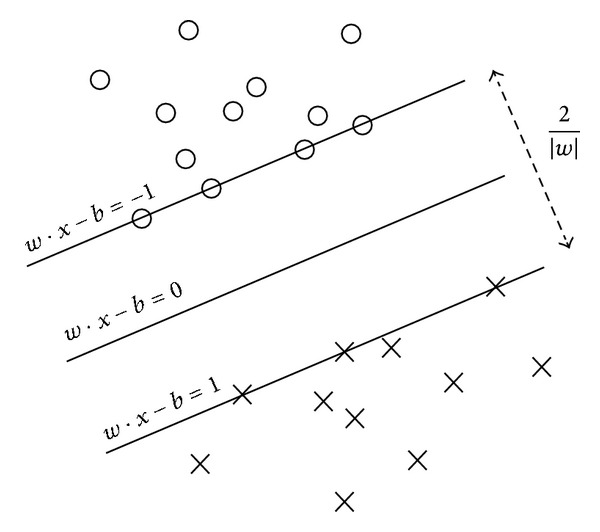
Optimal hyperplane.

When applying SVM to nonlinear dataset, we need to define a feature mapping function *x* ↦ *ϕ*(*x*). The feature mapping function is called kernel function. In the feature space, optimal hyperplane (Figure 1) can be gotten.

There are three common kernel functions:

*polynomial kernel*

(7) $$\begin{array}{c} k\left(x_{i},x_{j}\right)={\left(x_{i}\cdot x_{j}+a\right)}^{b}, \end{array}$$

*radial basis kernel*

(8) $$\begin{array}{c} k\left(x_{i},x_{j}\right)=e^{-({||x_{i}-x_{j}||}^{2}/2\sigma^{2})}, \end{array}$$

*sigmoidal kernel *

(9) $$\begin{array}{c} k\left(x_{i},x_{j}\right)=\tanh\left(ax_{i}\cdot x_{j}-b\right), \end{array}$$

where *a* and *b* are parameters defining the kernel's behavior.

In order to use SVM to solve a classification or regression problem on dataset that is nonlinearly separable, we need to first choose a kernel and relevant parameters which you expect they might map the nonlinearly separable data into a feature space where it is linearly separable. This is more of an art than an exact science and can be achieved empirically, for example, by trial and error. Sensible kernels to start with are polynomial, radial basis, and sigmoid kernels.

For classification, we need the following.

Create *H*, where *H*_*ij*_ = *y*_*i*_ *y*_*j*_ *ϕ*(*x*_*i*_)*ϕ*(*x*_*j*_).

Choose how significantly misclassifications should be treated, by selecting a suitable value for the parameter *C*.

Find *α* so that

(10)  max⁡α [∑i=1Lαi−12αTHα] s.t. 0≤αi≤C, ∑i=1Lαiyi=0.

Calculate

(11) $$\begin{array}{c} w=\overset{L}{\underset{i=1}{\sum }}\alpha_{i}y_{i}\phi \left(x_{i}\right). \end{array}$$

Determine the set of support vectors *V*, by finding the indices such that 0 ≤ *α*_*i*_ ≤ *C*.

Calculate

(12) $$\begin{array}{c} b=\frac{1}{N_{v}}\overset{}{\underset{v\in V}{\sum }}\left(y_{v}-\overset{}{\underset{i\in V}{\sum }}\alpha_{i}y_{i}\phi \left(x_{i}\right)\phi \left(x_{v}\right)\right). \end{array}$$

Each new point *x*′ is classified by evaluating *y*′ = sgn⁡(*wϕ*(*x*′) + *b*).

#### 2.1.2. PSO-SVM

Particle swarm optimization is an evolutionary computation technique proposed by Kennedy and Eberhart. It is a population-based stochastic search process, modeled after the social behavior of a bird flock [11, 12]. It is similar in spirit to birds migrating in a flock toward some destination, where the intelligence and efficiency lie in the cooperation of an entire flock [13]. PSO algorithms make use of particles moving in an *n*-dimensional space to search for solutions for *n*-variable function optimization problem. All particles have fitness values which are evaluated by the fitness function to be optimized and have velocities which direct the flying of the particles. The particles fly through the problem space by following the particles with the best solutions so far. PSO is initialized with a group of random particles (solutions) and then searches for optima by updating each generation [14].

SVM also has a drawback that limits the use of SVM on academic and industrial platforms: there are free parameters (SVM hyperparameters and SVM kernel parameters) that need to be defined by the user. Since the quality of SVM regression models depends on a proper setting of these parameters, the main issue for practitioners trying to apply SVM is how to set these parameter values (to ensure good generalization performance) for a given training dataset.

SVM based on PSO optimizes two important hyperparameters *C* and *ε* using PSO. The hyperparameter *C* determines the trade-off between the model complexity and the degree to which deviations larger than *ε*are tolerated. A poor choice of *C* will lead to an imbalance between model complexity minimization (MCM) and empirical risk minimization (ERM). The hyperparameter *ε* controls the width of the *ε*-insensitive zone, and its value affects the number of SVs used to construct the regression function. If *ε* is set too large, the insensitive zone will have ample margin to include data points; this would result in too few SVs selected and lead to unacceptable “flat” regression estimates [15].

#### 2.1.3. Cuckoo Search [16]

Cuckoo search is an optimization algorithm developed by Xin-she Yang and Suash Deb [16–18]. It was inspired by the obligate brood parasitism of some cuckoo species by laying their eggs in the nests of other host birds (of other species). Some host birds can engage direct conflict with the intruding cuckoos. For example, if a host bird discovers that the eggs are not their own, it will either throw these alien eggs away or simply abandon its nest and build a new nest elsewhere. Some cuckoo species such as the New World brood-parasitic* Tapera* have evolved in such a way that female parasitic cuckoos are often very specialized in the mimicry in colors and pattern of the eggs of a few chosen host species [19].

Cuckoo search idealized such breeding behavior and thus can be applied for various optimization problems. It seems that it can outperform other metaheuristic algorithms in applications [20].

Cuckoo search (CS) uses the following representations.

Each egg in a nest represents a solution, and a cuckoo egg represents a new solution. The aim is to use the new and potentially better solutions (cuckoos) to replace a not-so-good solution in the nests. In the simplest form, each nest has one egg. The algorithm can be extended to more complicated cases in which each nest has multiple eggs representing a set of solutions.

CS is based on three idealized rules:

1. each cuckoo lays one egg at a time and dumps its egg in a randomly chosen nest;
2. the best nests with high quality of eggs will be carried over to the next generation;
3. the number of available hosts nests is fixed, and the egg laid by a cuckoo is discovered by the host bird with a probability *P*_*a*_ ∈ (0,1). Discovering operate on some set of worst nests, and discovered solutions dumped from farther calculations.

In addition, Yang and Deb discovered that the random-walk style search is better performed by Lévy flights rather than simple random walk.

The pseudocode can be summarized as in Algorithm 1.

An important advantage of this algorithm is its simplicity. In fact, compared with other population- or agent-based metaheuristic algorithms such as particle swarm optimization and harmony search, there is essentially only a single parameter *P*_*a*_ in CS (apart from the population size *n*). Therefore, it is very easy to be implemented.

#### 2.1.4. CS-PSO-SVM

CS-PSO-SVM consists of two stages: firstly, a CS based approach for parameter optimization of SVM is developed to find the better initial parameters of kernel function and then PSO is applied to continue SVM training and find the best parameters of SVM. CS-PSO-SVM algorithm can be shown as follows in detail.

**Step 1**

Initializing cuckoo and PSO with population size, inertia weight, generations, and the range of hyperparameters *C* and *ε*.

**Step 2**

Applying CS to find the better initial parameters *C* and *ε*.

**Step 3**

Evaluating the fitness of each particle.

**Step 4**

Comparing the fitness values and determining the local best and global best particle.

**Step 5**

Updating the velocity and position of each particle till the value of the fitness function converges.

**Step 6**

After converging, the global best particle in the swarm is fed to SVM classifier for training.

**Step 7**

Training and testing the SVM classifier.

**Figure 2:**
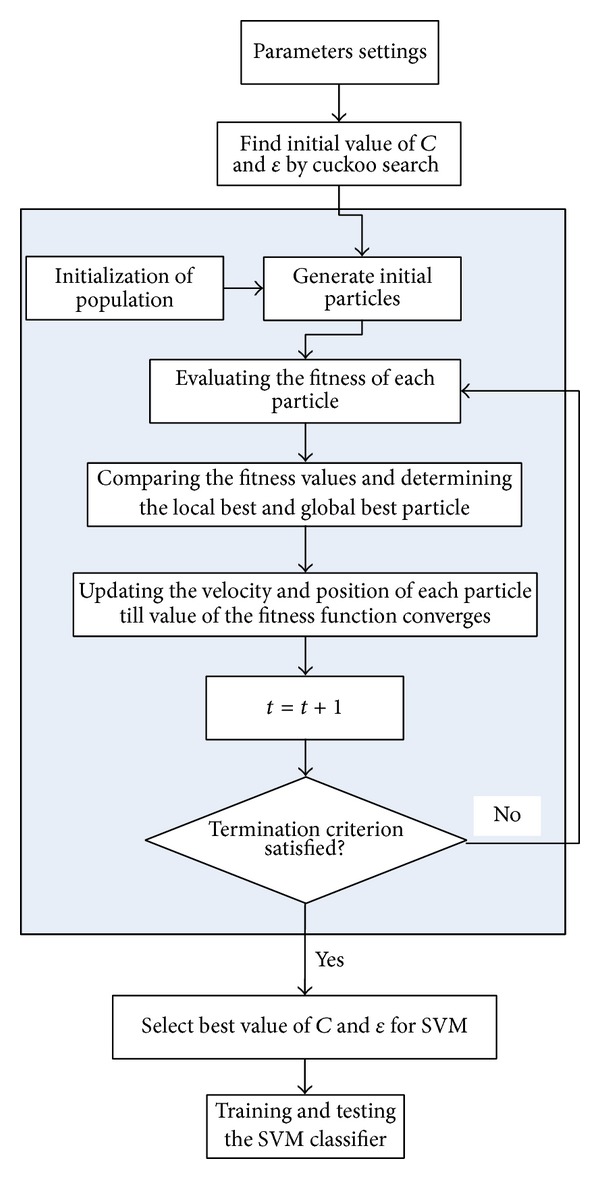
The flowchart of CS-PSO-SVM.

The flowchart of CS-PSO-SVM algorithm is shown in Figure 2 in detail.

### 2.2. Materials

In this study, numerical experiments use two datasets, heart disease dataset, and breast cancer dataset from UCI Machine Learning Repository [21].

Statlog (heart) dataset has 270 instances. In this dataset, there are thirteen numerical attributes, including age, sex, chest, blood (Rbp), serum (mg/dL), sugar (Fbs), electrocardiographic (ECG), heart rate, angina, old peak, slope, vessels (0–3), and THAL. Number of the presence of heart disease in the patient is 150, and number of the absence of heart disease in the patient is 120. Breast cancer dataset has 699 instances. Number of attributes of each instance is nine. There are thirteen numerical attributes including radius (mean of distances from center to points on the perimeter), texture (standard deviation of gray-scale values), smoothness (local variation in radius lengths), compactness, concavity (severity of concave portions of the contour), concave points (number of concave portions of the contour), symmetry, and fractal dimension. There are 458 as “benign” and 241 as “malignant.” In the above two datasets, “1” denotes one people as “benign” and “2” denotes one people as “malignant.” Table 1 shows the detail of two datasets. Heart disease dataset and breast cancer dataset choose 190 and 549 instances as train datasets, respectively. The rest of two datasets are test datasets. Continuous attributes are normalized firstly and then used to train and test.

## 3. Results and Discussion

**Figure 3:**
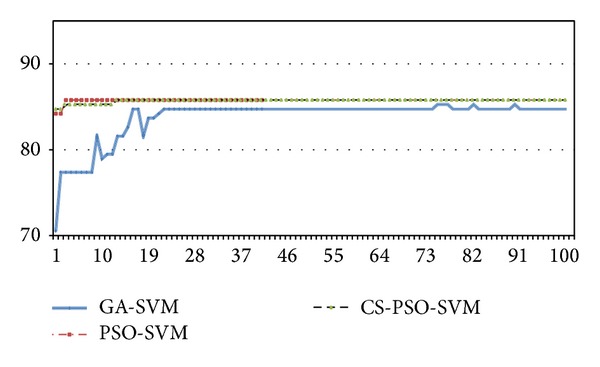
Population best fitness value in heart disease dataset.

**Figure 4:**
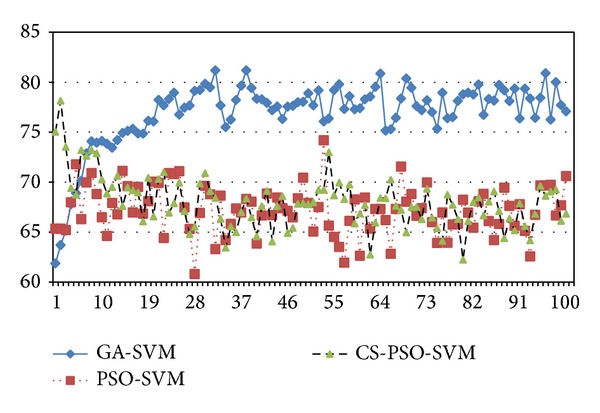
Population average fitness value in heart disease dataset.

**Figure 5:**
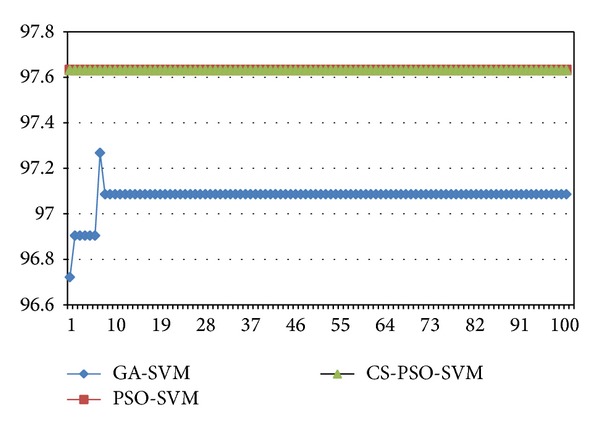
Population best fitness value in breast cancer dataset.

**Figure 6:**
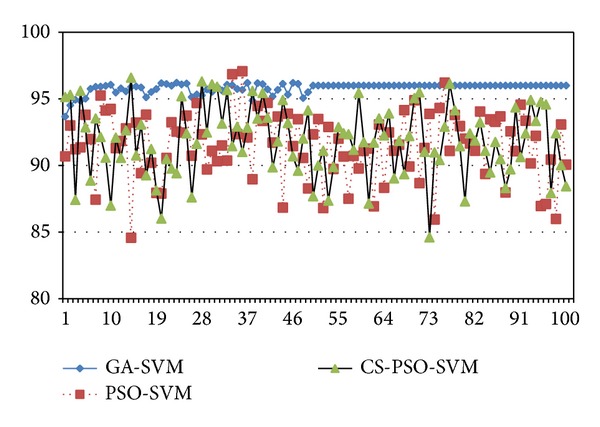
Population average fitness value in breast cancer dataset.

For the comparison of performance between traditional GA-SVM, PSO-SVM, and CS-PSO-SVM, these models are run several times. The program of the new algorithm is written by MATLAB 2012b and run on a computer with 2.0 GHz CPU, 1 GB DDR RAM. Figures 3 and 5 show the different curves of population best fitness value in heart disease dataset and breast cancer dataset by GA-SVM, PSO-SVM, and CS-PSO-SVM. Figures 4 and 6 show the different curves of population average fitness value in heart disease dataset and breast cancer dataset by the above three algorithms.  Table 2 lists the appropriate values of these parameters in three algorithms.

Table 3 shows the accuracy comparison of GA-SVM, PSO-SVM, and CS-PSO-SVM. For the heart disease dataset, in the test subset, the accuracy of CS-PSO-SVM is 85% and PSO-SVM and GA-SVM are 80%. In the training subset, the accuracy of CS-PSO-SVM is 100%.

For the breast cancer dataset, in the test subset, the accuracy of CS-PSO-SVM is 91.333% and PSO-SVM and GA-SVM are 90%. The results of empirical analysis showed that the predictive ability of all the models is acceptable. However, the CS-PSO-SVM results outperformed the other methods. Therefore, CS-PSO-SVM is a more effective model to predict disease in two datasets.

## 4. Conclusion

In the last few decades, several disease diagnosis models have been developed for the disease prediction. The objective of disease diagnosis models is to make a definite diagnosis from patients' laboratory sheet early as soon as possible and initiate timely treatment. Accurate diagnosis has an important meaning for human health. In this paper, we design a new disease diagnosis model, CS-PSO-SVM. The experimental research results show that the novel algorithm is better than GA-SVM and PSO-SVM.

## Figures and Tables

**Algorithm 1:**
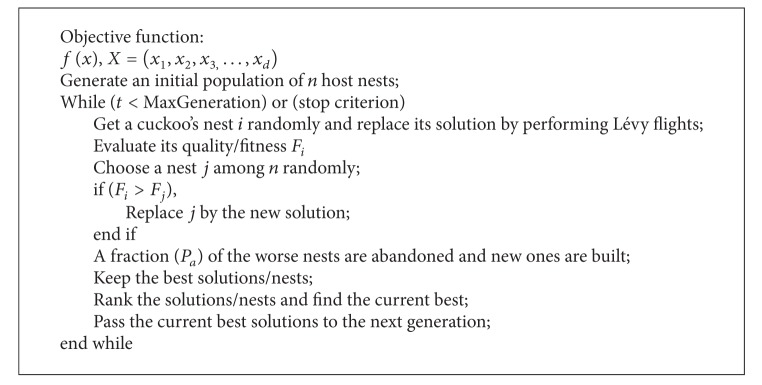
titleworktilte

**Table 1:** Credit dataset.

Dataset	Number of instances	Number of attributes	Benign	Malignant
Heart disease dataset	270	13	150	120
Breast cancer dataset	699	9	458	241

**Table:** (a)

Parameter	PopSize	Iteration	*p*_*c*_	*p*_*m*_
Value	20	100	0.5	0.005

**Table:** (b)

Parameter	PopSize	Iteration	*c*_1_	*c*_2_
Value	20	100	1.5	1.7

**Table:** (c)

Parameter	PopSize	Iteration	*P*_*a*_	PSO-*c*_1_	PSO-*c*_2_
Value	20	100	0.25	1.5	1.7

**Table 3:** Comparison of models.

Dataset	Algorithm	Forecasting accuracy total in training set	Forecasting accuracy total in test set	Precision	Recall	*F*-measure
Heart disease dataset	GA-SVM	85.7895%	80%	76.09%	87.50%	81.40%
	PSO-SVM	86%	80%	76.09%	87.50%	81.40%
	CS-PSO-SVM	**100%**	**85%**	**81.82%**	**90.00%**	**85.71%**

Breast cancer dataset	GA-SVM	**99.8179%**	90.0%	**92.08%**	62.00%	74.10%
	PSO-SVM	97.4499%	90.0%	88.99%	**64.67%**	74.90%
	CS-PSO-SVM	98.3607%	**91.3333%**	91.43%	64.00%	**75.29%**
